# SARS-CoV-2 and Implantation Window: Gene Expression Mapping of Human Endometrium and Preimplantation Embryo

**DOI:** 10.3390/life11121378

**Published:** 2021-12-10

**Authors:** Delphine Haouzi, Frida Entezami, Edward Tuaillon, Anna Gala, Alice Ferrières-Hoa, Sophie Brouillet, Alain R. Thierry, Samir Hamamah

**Affiliations:** 1 Univ Montpellier, INSERM U1203, DEFE, 34295 Montpellier, France; delphine.haouzi@inserm.fr (D.H.); Frida.ENTEZAMI@ahparis.org (F.E.); a-gala@chu-montpellier.fr (A.G.); a-ferrieres@chu-montpellier.fr (A.F.-H.); s-brouillet@chu-montpellier.fr (S.B.); 2 IRMB (Institute for Regenerative Medicine & Biotherapy), Univ Montpellier, INSERM, 34295 Montpellier, France; 3 CHU Montpellier, ART/PGD Department, Arnaud de Villeneuve Hospital, 34295 Montpellier, France; 4 Global ART Innovation Network, IRMB, CHU Montpellier, 34295 Montpellier, France; 5 ART Department, American Hospital of Paris, 92200 Neuilly-Sur-Seine, France; 6 CHU Montpellier, Bacteriology-Virology Department, 34295 Montpellier, France; e-tuaillon@chu-montpellier.fr; 7 Regional Institute of Cancer of Montpellier, 34090 Montpellier, France; alain.thierry@inserm.fr

**Keywords:** SARS-CoV-2, human, endometrial receptivity, preimplantation embryos

## Abstract

Understanding whether SARS-CoV-2 could infect cells and tissues handled during ART is crucial for risk mitigation, especially during the implantation window when either endometrial biopsies are often practiced for endometrial receptivity assessment or embryo transfer is performed. To address this question, this review analyzed current knowledge of the field and retrospectively examined the gene expression profiles of SARS-CoV-2-associated receptors and proteases in a cohort of ART candidates using our previous Affymetrix microarray data. Human endometrial tissue under natural and controlled ovarian stimulation cycles and preimplantation embryos were analyzed. A focus was particularly drawn on the renin-angiotensin system, which plays a prominent role in the virus infection, and we compared the gene expression levels of receptors and proteases related to SARS-CoV-2 infection in the samples. High prevalence of genes related to the *ACE2* pathway during both cycle phases and mainly during the mid-secretory phase for *ACE2* were reported. The impact of COS protocols on endometrial gene expression profile of SARS-CoV-2-associated receptors and proteases is minimal, suggesting no additional potential risks during stimulated ART procedure. In blastocysts, *ACE2*, *BSG*, *CTSL*, *CTSA* and *FURIN* were detectable in the entire cohort at high expression level. Specimens from female genital tract should be considered as potential targets for SARS-CoV-2, especially during the implantation window.

## 1. Introduction

SARS-CoV-2 infection has been surprising by its symptoms, sometime atypical, and ranging from minimal forms to life-threatening distress. In addition to the lungs, many other organs can be affected, including the heart, kidney, liver, gastrointestinal tract as well as the male gonadal function [1,2,3]. Therefore, identification of potential organ/cell targets for SARS-CoV-2 infection presents a challenge during the pandemic, especially in the scarcely explored field of assisted reproductive technologies (ART). To address this issue, some recent studies focused on the transcriptomic and/or proteomic profiling of SARS-CoV-2-associated receptors and proteases related to the virus infection in different human tissues [4]. In brief, SARS-CoV-2 entry requires the binding of its spike (S) glycoprotein to the host cell receptor angiotensin-converting enzyme 2 (*ACE2*) before it’s cleavage by cellular proteases such as *TMPRSS2* (transmembrane serine protease 2), *CTSL* (cathepsin L) or *FURIN* [5,6]. *ACE2* is part of the renin-angiotensin system (RAS) and clinical evidence suggests the occurrence of RAS imbalance in severe cases of SARS-CoV-2 infection [7,8]. Other cell surface proteins, including *BSG* (basigin) and *ANPEP* (analyl aminopeptidase membrane), have been recently reported to promote SARS-CoV-2 entry into target cells [9,10]. However, most studies have focused their analyses mainly on *ACE2* and *TMPRSS2* expression, resulting in very partial knowledge, especially in the female genital tract [1,11,12]. Moreover, it was recently reported that *ACE2* is required for the decidualization process in primary human endometrial stromal cells [13], a necessary step for the endometrial receptivity acquisition and successful implantation. To date, no studies have analyzed the potential impact of COS protocols during ART procedure on the gene expression profiles of SARS-CoV-2-associated receptors and proteases in parallel in human endometrial tissues and in preimplantation embryos during the implantation window. Therefore, as during ART, various cells and tissues, such as embryos and endometrial tissues, are handled and/or manipulated in the laboratory for the purpose of treatment or diagnosis; the investigation of SARS-CoV-2 host entry candidates in endometrial tissues from patients recruited for endometrial receptivity appreciation during the theoretical implantation window using the win-Test [14], and in preimplantation embryos, deserves investigation.

Hence, the aim of our review was to shed light on the susceptibility of cells and tissues manipulated during ART procedures to SARS-CoV-2 infection by analyzing (i) data from published reports, and our own previous transcriptomic data (ii) to report the gene expression profiles of SARS-CoV-2 host entry candidates in endometrial tissues, in both natural and stimulated cycles from patients undergoing endometrial receptivity assessment using the win-Test, with a focus on the renin-angiotensin system (RAS), and (iii) the gene expression levels of certain receptors (*ACE2*, *BSG)* and proteases (*TMPRSS2*, *CTSL*, *CTSA*, *FURIN*) closely related to the SARS-CoV-2 infection in human preimplantation embryos. To our knowledge this is the first time that data were analyzed concurrently in endometrium and preimplantation embryos.

## 2. Methods

All samples were part of our previous studies, which received approval from the Institutional Review Board of the Montpellier University Hospital and written informed consent from patients. For endometrium samples, the study population included 31 patients referred for ICSI for male infertility factor [15]. All normo-responder non COVID-19 patients had two endometrial biopsies within the same natural menstrual cycle during the early- (31 samples) and mid-secretory (31 samples) phase [15]. Twenty-one of the thirty-one patients were also evaluated in a subsequent stimulated cycle at the same menstrual phases (early- and mid-secretory phase) as described earlier [16]. Transcriptomes of human blastocystes (*n* = 10) and trophectoderm cells (*n* = 5) were obtained from our previously described microarray data [17,18]. RNA extraction and microarray data processing were also previously explained [15,16,17,18]. In brief, labeled fragmented complementary RNA was hybridized to oligonucleotide probes on Affymetrix HG-U133 Plus 2.0 arrays. Probe intensities were derived using the MAS5.0 algorithm to determine whether a gene is expressed with a defined confidence level or not (detection call). This call can either be ‘present’ (perfect match probes significantly more hybridized than the mis-match probes, false discovery rate (FDR) < 0.04) or ‘absent’ (FDR > 0.06). We used the GSEA software to download list of genes related to the KEGG-renin-angiotensin system (RAS) (https://www.gsea-msigdb.org/gsea/msigdb/cards/KEGG_RENIN_ANGIOTENSIN_SYSTEM.html; accessed on 11 May 2020). For each gene, the number of patients or samples with the probe set ‘present’, based on the detection call, was analyzed. For each probe set, the median of the signal intensities between samples was calculated and then, based on the median signal distribution, three groups were labelled as low (<100), medium (100–200) or high expression level (>200). Hierarchical clustering analyses based on the median expression levels per group were performed with the CLUSTER and TREEVIEW software packages. Then, according to the documented mechanisms of SARS-CoV-2 infection, the requirement for dual expression of specific receptors (*ACE2*, *BSG*) and proteases (*TMPRSS2*, *CTSL*, *CTSA*, *FURIN*), necessary for virus entry into target cells, were analyzed. Finally, for probe set present in at least four samples per group (early- vs. mid-secretory phase), differences in gene expression profile between menstrual phases were evaluated. Groups were compared using the Wilcoxon matched-pairs signed rank test, *t* test or Mann–Whitney test. Statistical analyses were performed using the GraphPad Prism 8.2.1 software. 

## 3. Results

### 3.1. Endometrial Receptivity Assessment Using the Win-Test during the Implantation Window

The endometrial receptivity assessment is under investigation during ART practice, especially in patients with repeated implantation failures (RIF), for whom it was shown to be useful in some reports. These studies somehow need validation by prospective randomized clinical trials to confirm the preliminary results. Several molecular diagnostic tools are available to synchronize the embryo maturity with the endometrial receptivity status, a mandatory condition for successful embryo implantation. Our transcriptomic approach associated to a meta-analysis of all reported transcriptomic studies comparing the same endometrial dating during natural cycle (early vs. mid-secretory stages), led us to define a specific molecular signature of human endometrial receptivity in natural cycle [15,16,19]. From this signature, a set of genes have been selected to develop a diagnostic tool based on quantitative RT-PCR: the ‘win-test’ (window implantation-test) [14,20]. These specific biomarkers of human endometrial receptivity allow one to classify an endometrial sample obtained during the implantation window as ‘receptive’, ‘partially receptive’ or ‘non-receptive’. The win-test strategy determines the specific cycle day when the endometrium is branded as receptive during the implantation window, in order to customize the timing of a subsequent embryo transfer and to synchronize the embryo stage with the endometrial receptivity status [14]. Based on the expression levels of our selected set of genes related to the endometrial receptivity, the win-test prediction for successful implantation (positive β-hCG) using receiver operating characteristics analysis revealed a specificity and sensitivity of 63.3% and 71%, respectively [14]. To date, 3030 biopsies performed during the implantation window from 1473 patients under natural cycle or hormone replacement therapy (HRT) protocol have been screened with the win-test in a mock frozen embryo transfer cycle. Patients underwent serial endometrial biopsies through the presumed implantation window, i.e., 6 to 9 days after the spontaneous LH surge for those on natural cycle, or 5 to 9 days after the onset of progesterone administration for those on HRT. Endometrial biopsies were repeated until the identification of the specific cycle day within the implantation window, where endometrium was labelled as receptive. The win-test screening has highlighted that both the occurrence time and the duration of the receptivity window are patient-dependent, confirming that identification of the optimal timing for embryo transfer for each patient is essential to optimize the effectiveness of ART and to achieve a successful pregnancy. The clinical effectiveness of the win-test strategy was first evaluated in RIF patients by a prospective non-randomized interventional multicenter study [14]. Whatever the embryo stage (cleavage stage embryos vs. blastocyst), the clinical pregnancy rate per patient was significantly higher after frozen embryo transfer according to the win-test strategy compared to the usual procedure for timing the embryo transfer (31.3 vs. 11.4%, *p* = 0.0004), increasing the live birth rate in RIF patients by a factor of four [14]. A prospective study in patients before their first or second attempt (mean ± SD, number of previous failed attempts: 1.2 ± 1) is under investigation and preliminary results in 38 non-RIF patients were very promising. Forty-nine percent (*n* = 19), 24% (*n* = 9), 15% (*n* = 6), and eleven percent (*n* = 4) of patients were referred for female, male, idiopathic and mixed infertility, respectively (mean ± SD, age: 36.3 ± 5.4 years). More precisely, 63% (*n* = 12), 16% (*n* = 3), 16% (*n* = 3) and 5% (*n* = 1) of female infertility were related to a diminished ovarian reserve in advanced female age, tubal factor, polycystic ovary syndrome and other (homosexual woman in sperm donation program), respectively. In this cohort of patients, with a mean of previous failed attempts of 1.2 ± 1, 55.3% and 41.3% of clinical pregnancy and live birth rates per patient were obtained, respectively, with a mean (±SD) of 1.2 ± 0.6 transferred embryos per cycle. However, the highest pregnancy rates with the win-test were obtained in oocyte/embryo donation program as reported in our preliminary results in 45 patients (mean ± SD, age: 41.2 ± 3.8 years) referred for female infertility due to an advanced female age (*n* = 36, age: 42.6 ± 2.5 years) or premature ovarian failure (*n* = 9, age: 35.5 ± 1.8 years), where 60 and 48.9% clinical pregnancy and live birth rates were achieved per patient. 

### 3.2. Endometrial SARS-CoV-2-Associated Receptors and Proteases during the Implantation Window under Natural and Stimulated Cycles

Several genes related to the RAS pathway were detected in endometrial samples of infertile patients undergoing ART. A higher prevalence was observed for *AGT*, *AGTR1*, *ANPEP*, *CTSA*, *ENPEP*, *LNPEP*, *MME*, *NLN* and *THOP1* at early- and mid-secretory cycle phase under both natural and stimulated cycles. The *ACE2* mRNA was detectable in endometrium from 42% to 52% of patients during the early secretory phase in natural and stimulated cycle respectively, while this rate reached 81% during the mid-secretory phase, whatever the cycle type (Table 1).

**Figure 1:**
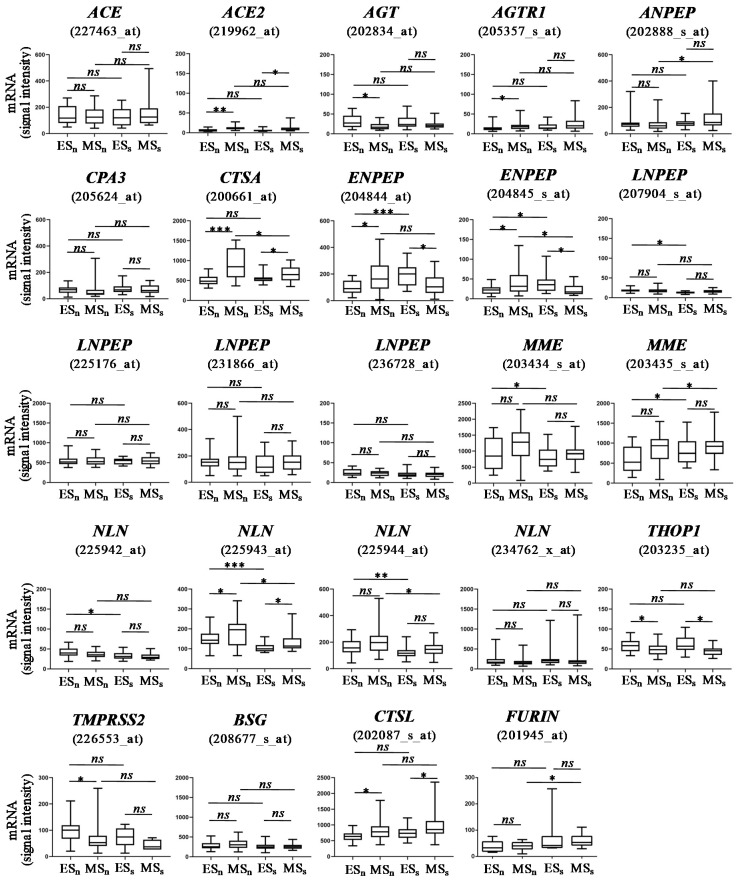
Gene expression level intensity in endometrium. Data are expressed as median [min; max] and statistical significance is analyzed by *t* test or Mann–Whitney test: ***, *p* ≤ 0.0001; **, *p* ≤ 0.001; *, *p* ≤ 0.05. ES_n_, natural early-secretory phase; ES_s_, stimulated early-secretory phase; MS_n_, natural mid-secretory phase; MSs, stimulated mid-secretory phase; ns, not significant.

The *ACE2* intensity signal was significantly over-expressed during the mid-secretory phase compared with the early-secretory phase under both natural and stimulated cycle, although the expression level was low (Figure 1). 

The analysis of *TMPRSS2* expression revealed a moderate prevalence during both phases, whatever the cycle type, with a medium gene expression level. Moreover, under natural cycle, the *ACE2*-*TMPRSS2* co-expression was found in 52% of patients (16/31) corresponding to 32% of endometrial samples (20/62) (Table 2). 

The prevalence of the *BSG* mRNA presence was high in both cycle phases, with a strong expression level. Finally, the *CTSL* was detectable in all endometrial samples, in both menstrual phases and cycle types, showing a high expression level. The most representatives of dual co-expression of SARS-CoV-2-associated receptor and protease in endometrium were *BSG*-*CSTL* and *BSG*-*CTSA* (Table 2). In addition, high intra-patient variability was observed for *ACE*, *ANPEP*, *CPA3*, *CTSA*, *ENPEP*, *LNPEP*, *MME*, *NLN*, *TMPRSS2* and *BSG* (Figure 1). 

### 3.3. SARS-CoV-2-Associated Receptors and Proteases in Human Preimplantation Embryos

In blastocysts, *ACE2*, *BSG*, *CTSL*, *CTSA* and *FURIN* were detectable in the entire cohort at high expression level (Table 3). Therefore, the prevalence of the different dual co-expression of SARS-CoV-2-associated proteases and receptors was optimal (100% of samples) (Table 2). Interestingly, only *CTSL* was detectable in all trophectoderm samples and a prevalence of 60% was found for the *BSG*-*CTSL* co-expression.

## 4. Discussion

The SARS-CoV-2 infection of the female genital tract is uncertain, especially for the endometrium. Understanding whether SARS-CoV-2 has the capacity to infect endometrium is crucial for considering the potential risks during ART procedure and for pregnancy. The present study aimed to assess the expression level of genes related to the angiotensin-converting enzyme 2 (*ACE2*) pathway and other alternative pathways (*TMPRSS2*, *BSG*, *CTSL, CTSA*, *FURIN*), known as targets for SARS-CoV-2 cell entry [6,10,21]. So far, one study reported the expression of *ACE2* mRNA in human proliferative and secretory endometrium in a limited number of samples [22]. Alongside a recent study analyzing the endometrial gene expression profile of certain targets for SARS-CoV-2 cell entry, including *TMPRSS2*, *BSG*, *CTSL*, *CTSA*, *FURIN* throughout the natural menstrual cycle [23], our study focused on genes related to the *ACE2* pathway in addition to SARS-CoV-2 targets, under both natural and stimulated cycles in the same patients. Indeed, using our large cohort of endometrial samples (*n* = 62) from 31 patients, we reported for the first time a high prevalence of genes related to the *ACE2* pathway, including *AGT*, *AGTR1*, *ANPEP*, *CTSA*, *ENPEP*, *LNPEP*, *MME*, *NLN*, *THOP1*, *BSG* and *CTSL* during both phases (early- and mid-secretory phase). In addition, *ACE2* was significantly over-expressed during the mid-secretory phase compared with the early-secretory phase, as reported [13,23], and was confirmed so, whatever the cycle type (natural and stimulated). This is consistent with a recent study which demonstrated that both *ACE2* mRNA and protein were over-expressed in stromal cells in the secretory phase and that *ACE2* is required for the stromal decidualization process [13]. The highest signal intensities were found for *CTSA*, *LNPEP*, *MME*, *NLN*, *BSG* and *CTSL*. These findings were in agreement with the study by Henarejos-Castillo et al. [23] showing a high mRNA expression level of *BSG* and *CTSL* while *ACE2* mRNA was low in endometrium. Overall, the impact of ovarian stimulation protocols on endometrial gene expression profile of SARS-CoV-2-associated receptors and proteases of infertile patients is minimal, suggesting no additional potential risk of SARS-CoV-2 infection during stimulated ART procedure compared with natural cycle. Our transcriptomic data strongly suggest that endometrial specimens should be considered as a potential target for SARS-CoV-2 in natural and stimulated cycles. However, these results should be interpreted cautiously without the proof of detection of SARS-CoV-2 nucleic acid in the endometrium. The correlation between the gene expression levels of receptors and proteases required for SARS-CoV-2 infection and the actual endometrial vulnerability to the virus has yet to be validated. In addition, the origin of the SARS-CoV-2 contamination of the tissues during ART procedure has not been reliably established [24]. Numerous contamination means may be suspected, such as by aerosol or sexual transmission [25]. Considering the endometrium as a direct target for the SARS-CoV-2 is somehow premature. Moreover, the hypothesis of SARS-CoV-2 infection changing the endometrial gene expression profile of its associated receptors and proteases leading to alteration of the endometrial receptivity status remain under question. Despite of the many issues unresolved to date, clinical data reported no increasing risk of pregnancy loss for patients with SARS-CoV-2 infection during the first pregnancy trimester compared to non-infected patients [26], suggesting no impact of SARS-CoV-2 on the endometrial receptivity status and successful implantation.

Our transcriptomic analyses revealed the abundant expression of *CTSL* protease, involved in the cleavage of the viral S protein, in human preimplantation embryos and in trophectoderm. Furthermore, *CTSA* and *FURIN* were abundant in blastocysts. Together, these findings strongly suggest that blastocysts are permissive to SARS-CoV-2 infection. Trophectoderm, the outer cell mass of the blastocyst that gives rise to trophoblast and the extra-embryonic structures, exhibited a moderate prevalence (60% of samples) of *BSG*-*CTSL* co-expression. This finding is consistent with current clinical data reporting a placental and neonates SARS-CoV-2 infection in pregnant women with symptomatic COVID-19 [27,28].

Based on transcriptomic data and literature review, analyses of genes expression related to the SARS-CoV-2-associated receptors and proteases suggest that the cells and tissues routinely handled and manipulated during ART procedures, especially the endometrium during the implantation window, are potential targets for viral infection. Performing the win-test to customize the timing of embryo transfer and therefore to optimize pregnancy outcome, during the COVID-19 pandemic requires extra precautions for endometrial biopsy process. Nevertheless, further studies are mandatory to validate the data at protein level, in ex vivo and in vivo models in order to confirm the susceptibility of the human preimplantation embryo and the endometrium to SARS-CoV-2 infection. In case the endometrial vulnerability is confirmed and the receptivity window altered, proper attention should be drawn on new generation COVID-19 vaccines that raise the concern of a massive production of the spike protein to trigger the antibodies. It could hence be assumed that the spike by itself may have the same consequences as the virus on the endometrium and embryo implantation and/or pregnancy. The still ongoing pandemic will help to shed light on these fundamental questions.

## Figures and Tables

**Table 1:** mRNA prevalence of genes potentially involved in the SARS-CoV-2 infection in endometrium during natural and stimulated cycles. The number of patients with genes ‘present’ is indicated for each cycle phase, as well as the percentage in brackets. Three housekeeping genes (*GAPDH*, *ACTB*, *STAT1*) are included as positive controls. *p* values are indicated when difference in mRNA prevalence between phases is significant. The expression level intensity is classified as low (+/−), medium (+), high (++) or absent (−). ns, not significant.

		Endometrial Tissus							
		Natural Cycle				Stimulated Cycle			
		ES Phase (*n* = 31)	MS Phase (*n* = 31)	Frequency Distribution		ES Phase (*n* = 21)	MS Phase (*n* = 21)	Frequency Distribution	
Gene Name	Gene ID	No. of Patients (%)	No. of Patients (%)	*p*-Value	Signal Intensity	No. of Patients (%)	No. of Patients (%)	*p*-Value	Signal Intensity
*ACE*	227463_at	19 (61.3)	16 (51.6)	ns	+	10 (47.7)	10 (47.7)	ns	+
*ACE2*	219962_at	13 (41.9)	25 (80.6)	0.0005	+/−	11 (52.4)	17 (80.9)	0.015	+/−
	222257_s_at	1 (3.2)	10 (32.3)	0.0009	+/−	2 (9.5)	10 (47.7)	0.002	+/−
*AGT*	202834_at	28 (90.3)	26 (83.9)	ns	+/−	21 (100)	19 (90.5)	ns	+/−
*AGTR1*	205357_s_at	22 (71.0)	30 (96.8)	0.008	+/−	19 (90.5)	21 (100)	ns	+/−
*AGTR2*	207294_at	11 (35.5)	4 (12.9)	0.02	+/−	1 (4.8)	0		+/−
*ANPEP*	202888_s_at	31 (100)	31 (100)	ns	+/−	21 (100)	21 (100)	ns	+
*CPA3*	205624_at	9 (29.0)	11 (35.5)	ns	+/−	16 (76.2)	14 (66.7)	ns	+/−
*CTSA*	200661_at	31 (100)	31 (100)	ns	++	21 (100)	21 (100)	ns	++
*ENPEP*	204844_at	30 (96.8)	30 (96.8)	ns	+/−	21 (100)	21 (100)	ns	+
	204845_s_at	28 (90.3)	29 (93.5)	ns	+/−	21 (100)	18 (85.7)	ns	+/−
*LNPEP*	207904_s_at	22 (71.0)	26 (83.9)	ns	+/−	12 (57.1)	11 (52.4)	ns	+/−
	225176_at	31 (100)	31 (100)	ns	++	21 (100)	21 (100)	ns	++
	231866_at	31 (100)	31 (100)	ns	++	21 (100)	21 (100)	ns	+
	236728_at	27 (87.1)	24 (77.4)	ns	+/−	21 (100)	17 (80.9)	0.036	+/−
*MME*	203434_s_at	31 (100)	31 (100)	ns	++	21 (100)	21 (100)	ns	++
	203435_s_at	30 (96.8)	29 (93.5)	ns	++	21 (100)	21 (100)	ns	++
*NLN*	224063_at	3 (9.7)	3 (9.7)	ns	+/−	2 (9.5)	3 (14.3)	ns	+/−
	225942_at	22 (71.0)	23 (74.2)	ns	+/−	13 (61.9)	12 (57.1)	ns	+/−
	225943_at	31 (100)	31 (100)	ns	++	21 (100)	21 (100)	ns	+
	225944_at	31 (100)	31 (100)	ns	++	21 (100)	21 (100)	ns	+
	234762_x_at	31 (100)	31 (100)	ns	++	21 (100)	21 (100)	ns	++
*THOP1*	203235_at	31 (100)	31 (100)	ns	+/−	21 (100)	20 (95.2)	ns	+/−
*TMPRSS2*	226553_at	19 (61.3)	14 (45.2)	ns	+/−	11 (52.4)	7 (33.3)	0.042	+/−
*BSG*	208677_s_at	29 (93.5)	30 (96.8)	ns	++	19 (90.5)	20 (95.2)	ns	++
*CTSL*	202087_s_at	31 (100)	31 (100)	ns	++	21 (100)	21 (100)	ns	++
*FURIN*	201945_at	16 (51.6)	19 (61.3)	ns	+/−	13 (61.9)	15 (71.4)	ns	+/−
*GAPDH*	M33197_M_at	31 (100)	31 (100)	ns	++	21 (100)	21 (100)	ns	++
*ACTB*	X00351_5_at	31 (100)	31 (100)	ns	++	21 (100)	21 (100)	ns	++
*STAT1*	M97935_3_at	31 (100)	31 (100)	ns	+/−	21 (100)	21 (100)	ns	+/−

**Table 2:** mRNA prevalence of dual co-expression of SARS-CoV-2-associated receptors and proteases involved in virus infection. The number of samples with genes ‘present’ is indicated for each sample group, as well as the percentage in brackets. Bl, blastocysts; TE, trophectoderm.

*ACE2-TMPRSS2*	*ACE2-BSG*	*ACE2-CTSL*	*ACE2-CTSA*	*ACE2-FURIN*	*BSG-TMPRSS2*	*BSG-CTSL*	*BSG-CTSA*	*BSG-FURIN*	
20 (32.3)	37 (59.7)	38 (61.3)	38 (61.3)	25 (40.3)	30 (48.4)	59 (95.2)	59 (95.2)	34 (54.8)	Endometrium (*n* = 62)
4 (40)	10 (100)	10 (100)	10 (100)	10 (100)	4 (40)	10 (100)	10 (100)	10 (100)	Bl (*n* = 10)
0	0	1 (20)	0	1 (20)	2 (40)	3 (60)	2 (40)	1 (20)	TE (*n* = 5)

**Table 3:** mRNA prevalence of genes potentially involved in the SARS-CoV-2 infection in human preimplantation embryos and trophectoderm samples. The number of samples with genes ‘present’ are indicated, as well as the percentage in brackets. Three housekeeping genes (*GAPDH*, *ACTB*, *STAT1*) are included as positive controls. The expression level intensity is classified as low (+/−), medium (+), high (++) or absent (−).

		Blastocyst		Trophectoderm	
Gene Name	Gene ID	No. of Samples (%)	Signal Intensity	No. of Samples (%)	Signal Intensity
*ACE2*	219962_at	10 (100)	++	1 (20)	+/−
	222257_s_at	10 (100)	++	2 (40)	+
*TMPRSS2*	226553_at	4 (40)	++	3 (60)	+
*BSG*	208677_s_at	10 (100)	++	3 (60)	+/−
*CTSL*	202087_s_at	10 (100)	++	5 (100)	++
*CTSA*	200661_at	10 (100)	++	2 (40)	+/−
*FURIN*	201945_at	10 (100)	+	2 (40)	+/−
*GAPDH*	M33197_M_at	10 (100)	++	5 (100)	++
*ACTB*	X00351_5_at	10 (100)	+	5 (100)	++
*STAT1*	M97935_3_at	10 (100)	+/−	5 (100)	+/−
